# Pathogen- and Host-Directed Antileishmanial Effects Mediated by Polyhexanide (PHMB)

**DOI:** 10.1371/journal.pntd.0004041

**Published:** 2015-10-02

**Authors:** Rebuma Firdessa, Liam Good, Maria Cecilia Amstalden, Kantaraja Chindera, Nor Fadhilah Kamaruzzaman, Martina Schultheis, Bianca Röger, Nina Hecht, Tobias A. Oelschlaeger, Lorenz Meinel, Tessa Lühmann, Heidrun Moll

**Affiliations:** 1 Institute for Molecular Infection Biology, University of Würzburg, Würzburg, Germany; 2 Royal Veterinary College, University of London, London, United Kingdom; 3 Institute for Pharmacy and Food Chemistry, University of Würzburg, Würzburg, Germany; University of Notre Dame, UNITED STATES

## Abstract

**Background:**

Cutaneous leishmaniasis (CL) is a neglected tropical disease caused by protozoan parasites of the genus *Leishmania*. CL causes enormous suffering in many countries worldwide. There is no licensed vaccine against CL, and the chemotherapy options show limited efficacy and high toxicity. Localization of the parasites inside host cells is a barrier to most standard chemo- and immune-based interventions. Hence, novel drugs, which are safe, effective and readily accessible to third-world countries and/or drug delivery technologies for effective CL treatments are desperately needed.

**Methodology/Principal Findings:**

Here we evaluated the antileishmanial properties and delivery potential of polyhexamethylene biguanide (PHMB; polyhexanide), a widely used antimicrobial and wound antiseptic, in the *Leishmania* model. PHMB showed an inherent antileishmanial activity at submicromolar concentrations. Our data revealed that PHMB kills *Leishmania major* (*L*. *major) via* a dual mechanism involving disruption of membrane integrity and selective chromosome condensation and damage. PHMB’s DNA binding and host cell entry properties were further exploited to improve the delivery and immunomodulatory activities of unmethylated cytosine-phosphate-guanine oligodeoxynucleotides (CpG ODN). PHMB spontaneously bound CpG ODN, forming stable nanopolyplexes that enhanced uptake of CpG ODN, potentiated antimicrobial killing and reduced host cell toxicity of PHMB.

**Conclusions:**

Given its low cost and long history of safe topical use, PHMB holds promise as a drug for CL therapy and delivery vehicle for nucleic acid immunomodulators.

## Author Summary

Intracellular pathogens are difficult to kill because their localization inside host cells provides protection against immunity and chemotherapies. Thus, successful treatment of diseases caused by intracellular pathogens may need combination therapies and effective delivery systems. Cutaneous leishmaniasis (CL) is caused by intracellular protozoan pathogens of the genus *Leishmania*. CL is a long established global problem, yet there are no suitable vaccine or chemotherapy options. We found that the well-tolerated cationic polymer PHMB is able to directly kill *Leishmania major* (*L*. *major)* and to enhance host-directed killing by improving the delivery of immunomodulatory nucleic acids. The study exemplifies parallel host- and pathogen-directed killing of an intracellular pathogen in the presence of effective drug delivery platforms. This general strategy holds great promise for therapy of a range of diseases caused by intracellular pathogens.

## Introduction

Leishmaniasis has persisted for centuries as a life-threatening and disfiguring disease, endemic to 98 countries across the globe, with an overall estimated prevalence of 12 million and a yearly incidence of 2 million new cases [1,2]. It mainly affects the poorest regions of the world, where patients cannot afford medication. It is also endemic in several developed countries including France and Spain, and there are reports of increasing cases of leishmaniasis in developed countries, where the disease is considered non-endemic [3,4]. Leishmaniasis is classically subdivided into three main clinical forms: cutaneous, mucocutaneous, and visceral. Two thirds of all cases worldwide are cutaneous leishmaniasis (CL) presentations [2,5,6]. CL symptoms range from single, self-healing cutaneous wounds to a persistent, metastatic disease [7]. The basis for such diverse pathologies is multifactorial and complex, and innate immune system functioning and its pattern recognition receptors are determining factors [7,8]. Thus, host immunity is a decisive factor that influences the outcome of *Leishmania* infection. Furthermore, *Leishmania* parasites manipulate and subvert host immune responses. For example, *L*. *major* infection shifts cellular immunity, associated with cytokines such as interleukin (IL)-12, interferon gamma (IFN-γ) and tumor necrosis factor (TNF)-α producing Th1 CD4^+^ T lymphocytes, to humoral immunity, associated with (Th2) CD4^+^ T lymphocytes responses in susceptible hosts [9–11]. Conversely, the host immune deviation towards Th1 results in effective removal of the parasite from the host, and is a promising approach for intracellular pathogen therapy.

CL is a neglected tropical disease, and medicine does not provide a suitable therapy. The available treatments are old and the systemic side effects often outweigh any clinical benefits [12,13]. After decades of research in drug development, there is still no new commercial drug for treatment of CL. Most patients in the affected regions are poor and cannot afford medication, discouraging pharmaceutical industry investment. Alternative treatment approaches that are safe, effective and readily accessible to third-world countries are of major interest. Among several drug discovery strategies, repositioning existing drugs from other areas of disease is considered to be a cost-effective strategy and accounts for many currently used anti-parasitic drugs, and thus, has historically played an important role in anti-parasitic drug development [14]. For example, amphotericin B, miltefosine and paromomycin have all been repositioned to treat leishmaniases.

The current treatment regimens against CL can be either local or systemic. The choice of systemic versus local therapy is based on the causative *Leishmania* subspecies, geographic regions, severity of the diseases and the patient’s immune status [15,16]. Complex cases such as patients having more than three lesions, singular lesion measuring > 40 mm in diameter, lesion in cosmetically and functionally delicate body parts (such as face, joints, mucocutaneous zones, lymph nodes) and immunosuppressed patients should be treated systemically [16]. Parenteral administration of antimonials, pentamidine, amphotericin B or oral miltefosine is the standard systemic treatment against CL. However, their efficiencies are limited by several factors, including significant toxicity and other side effects [2,3,15,17]. Local treatment such as paromomycin ointment and infiltration with antimonials has been recommended by the World Health Organization (WHO) as a first line treatment for non-complicated CL cases [2]. Although local treatments offer significant advantages over systemic therapy, they have not yet displayed a strong and consistent effect. Overall, there is a lack of evidence for potential benefit of the current CL treatments. Insufficient killing of stealth parasites inside macrophages and insufficient drug concentrations within the dermis are factors that appear to hinder its clinical use [18,19], suggesting a need for improved drug delivery, absorption and retention strategies. Importantly, it has been shown that liposomes loaded with drugs enhance *in vitro* drug permeation across stripped skin and improves the *in vivo* antileishmanial activity in experimentally infected mice [12,20,21]. Also, modulation of the immune micro-environment for host-directed treatment against CL has been suggested as a potential therapeutic and prophylactic strategy [22]. Indeed, immune modulation towards Th1 responses using immune modulating agents such as CpG ODN showed promising efficacy against leishmaniasis [23–25]. A further advantage is that CpG ODN can accelerate wound repair [26,27]. Similarly, it has been shown that topical application of an immunomodulator imiquimod assists CL wound healing [21,28]. Therefore, an improved formulation of CpG ODN could stabilize oligomers against degradation, enhance cell uptake, increase killing within macrophages and aid wound repair.

PHMB is a synthetic cationic polymer recommended as a first-choice treatment for locally infected and critically colonized wounds [29]. PHMB has inherent antimicrobial effects and is used to treat certain parasite infections, but there are no reports of its use against intracellular pathogens or *Leishmania* species. As well as providing direct antimicrobial effects, PHMB may be able to mediate delivery of other therapeutics. Several synthetic and natural cationic polymers are used as nucleic acids carriers by forming polyplex (any complex of a polymer and nucleic acids) nanostructures through their ability to condense nucleic acids into particles, while allowing dissociation once inside the cell [30,31]. Therefore, PHMB has the potential for dual use against infections, providing both direct pathogen killing and enhanced delivery of nucleic acids that can modify the immune system to enhance killing.

Here we aimed to investigate the potential use of PHMB as an antileishmanial agent and as a non-viral nucleic acid carrier of CpG ODN. We found that PHMB itself has potent leishmanicidal activity at submicromolar concentrations. PHMB’s leishmanicidal mechanisms of action appear to involve disruption of parasite cell membranes, cell entry and condensation/disruption of parasite chromosomes. Also, we found that PHMB spontaneously binds CpG ODN and forms stable nanopolyplexes by interacting with CpG ODN at appropriate dose ratios. PHMB also protects and prompts cellular uptake of CpG ODN. Interestingly, the nanopolyplexes effectively counteract the toxicity associated with PHMB by interacting with negatively charged CpG ODN through charge complexation/shielding. Therefore, PHMB/CpG ODN can provide combined host- and pathogen-directed leishmanicidal effects against CL and may provide multiple benefits for CL therapy.

## Methods

### Cell culture

BMDM were generated from the bone marrow of BALB/c mice (Charles River Breeding Laboratories, Sulzfeld, Germany) and cultured for 6 days at 37°C and 5% CO_2_, as recently described [32]. The primary BMDM were then harvested and maintained in phenol-free complete Roswell Park Memorial Institute (RPMI) medium containing 10% fetal calf serum (heat-inactivated at 56°C for 30 min), 2 mM L-glutamine, 10 mM HEPES buffer, 0.05 mM β-mercaptoethanol solution, gentamicin (50 μg/ml) and penicillin G (100 u/ml) for all experiments. The same media were used in *L*. *major* parasites experiments. 293T kidney epithelial cells (DSMZ-No ACC-635, Braunschweig, Germany) were cultivated and maintained in high glucose (4.5 g/l) DMEM without L-glutamine and phenol red but containing 10% fetal calf serum (heat-inactivated), 200 mM L-glutamine and sodium pyruvate. Human adult calcium temperature (HaCaT, keratinocytes) were obtained from Dr. Amir Sharili, Queen Mary University of London. Keratinocytes were cultivated in DMEM containing 10% fetal bovine serum, with 4.5 g glucose/l, 0.11 g sodium pyruvate/L and L-glutamine 0.584 g/l.

### AlamarBlue assay

Cytotoxicities of the different compounds or formulations against mammalian cell (BMDM or 293T epithelial cells) and *L*. *major* promastigotes were determined as previously described [33]. Briefly, 4 × 10^4^ mammalian cells or 10 × 10^6^ virulent *L*. *major* promastigotes (MHOM/IL/81/FE/BNI strain) in the log phase of growth were added to each well of 96-well plates and cultured with five increasing concentrations of each compound at 37°C or 27°C for 24 h, respectively. Standard antileishmanial drugs including amphotericin B (A2942), pentamidine isothionate (P0547) and paromomycin sulfate (P9297) from Sigma-Aldrich (Deisenhofen, Germany) and miltefosine (1-hexadecylphosphorylcholine) (Cayman Chemical Company, Ann Arbor, MI, USA) were used as positive controls. The AlamarBlue dye (Trinova Biochem GmbH, Giessen, Germany) was added to each well at 10% concentration and incubated for a further 48 h. The optical density (OD) was then measured by using Multiskan Ascent ELISA reader at a wavelength 550 nm and 630 nm. The OD value or % dye reduction is proportional to viable cell/parasite number and was used for IC_50_ calculation based on the intercept theorem.

### Luciferase reporter assay (amastigote assay)

The assay is based on bioluminescence measurement of firefly luciferase, which catalyzes the formation of light from ATP and luciferin. The virulent luciferase-transgenic (Luc-tg) *L*. *major* strain containing a firefly luciferase reporter gene was maintained by continuous passage in female BALB/c mice and was grown in 96-well blood agar cultures at 27°C, 5% CO_2_, and 95% humidity. A luciferase-transgenic *L*. *major* strain was used to infect BMDM according to our recently established protocol [34]. Briefly, BMDM numbers were adjusted to 2 × 10^5^, cultured in 96-well plates and incubated for 4 h to allow adherence to the surface. The promastigote number was adjusted to 3 × 10^6^ parasites per ml to achieve an infection rate of 1:15. Then, infection was made by discarding the supernatant of the cultured cells and replacing it with an equal volume of complete RPMI medium containing the promastigotes' suspension. After incubating the infected BMDM (37°C, 5% CO_2_) for 24 h to allow full differentiation of the promastigotes into amastigotes, the medium containing the extracellular parasites was removed and the wells were washed three times with the same RPMI medium. Five serial dilutions of each substance (1:5) in the same fresh RPMI medium were added to the infected BMDM in duplicate with the appropriate controls. After further 24 h of incubation at 37°C and 5% CO_2,_ the infected BMDM were lysed with a luciferin-containing buffer (Britelite, PerkinElmer, Germany) and luminescence was measured with a luminescence plate reader, Victor X Light 2030 luminometer (PerkinElmer). The IC_50_ values of the compounds against intracellular amastigotes were calculated based on the intercept theorem, as previously described [34,35].

### Flow cytometry

PI and YO-PRO-1 dyes (Life Technologies, Darmstadt, Germany) uptake by the promastigotes was measured by MACS Quant Analyzer (Miltenyi Biotec, Bergisch Gladbach, Germany), and used to indicate membrane integrity [36]. YO-PRO-1 dye selectively passes through the plasma membranes of apoptotic cells but it does not label living cells [36]. *L*. *major* promastigotes were treated with 2 μM PHMB for indicated time points and then washed at 3,000 × g for 10 min. The samples were stained by incubating with PI or YO-PRO-1 dyes for 15 min in the dark at RT according to manufacturer’s instructions. The samples were then washed and a total of 100,000 events were immediately acquired by flow cytometry. Similarly, adherent BMDM cells were allowed to take-up fluorescent PHMB (PHMB-FITC conjugate) in the presence or absence of five different pharmacological inhibitors (cytochalasin D (C8273, used at 5 μg/ml), wortmannin (W1628, used at 12.85 μg/ml), chlorpromazine hydrochloride (C8138, used at 10 μg/ml), ikarugamycin (SML0188, used at 1 μM) and dynasore (D7693, used at 25.8 μg/ml), Sigma-Aldrich, Deisenhofen, Germany). PHMB (Polyhexamethylene biguanide; CAS# 27083-27-8; alternative chemical names: polyhexanide, polyamino propylbiguanide, M_w_ = 2780 g/mol) [37] was provided by the laboratory of Dr. Liam Good and by Tecrea Ltd, London UK (www.tecrea.co.uk), at stock concentrations of 1 mg/ml and 200 mg/ml. PHMB is a cationic and amphipathic structure composed of repeating basic biguanidine units connected by hexamethylene hydrocarbon chains. The structure and concentrations of PHMB as % weight/volume are shown in the supplementary information (S1 Fig and S1 Table). PHMB (PHMB-FITC) was produced according to recently developed method (further details will be provided in a separate publication, Chindera et al., submitted). Briefly: 2 mg of fluorescein isothiocyanate (FITC) was dissolved in dimethylformamide that contains 50 μl of N,N-diisopropylethylamine. The solution was then mixed with 50 mg of PHMB in 200 μl volume and shaken overnight at RT. The resulting mixture was dialyzed using molecular weight cut off membrane 3.5 kDa against 50% aqueous ethanol for 5 days with intermittent change of dialysate and lyophilized to obtain fluoresceinyl‐PHMB (PHMB‐FITC). Moreover, polyplexes were prepared between PHMB and 3’ end rhodamine red-labeled CpG ODN (CpG-R) (M_w_ = 6891 g/mol, sequence TCCATGACGTTCCTGATGCT, Eurofins MWG Operon, Germany) at 1:1 (w/w) ratio for uptake study. The poylplexes and CpG-R were incubated with BMDM in the absence or presence of the endocytosis inhibitors for various time points. The BMDM were harvested, washed, diluted to 1 × 10^6^ and a total of 20,000 events were acquired per sample by flow cytometry. The data were analyzed using FlowJo software (Tree Star Inc., CA, USA).

### Fluorescence microscopy and confocal microscopy

The BMDM or promastigotes were co-cultured with the indicated doses of fluorescent PHMB for 24 h or 48 h. The samples were transferred to 15-ml tubes, washed and fixed with 4% paraformaldehyde. The DNA of *L*. *major* promastigotes and BMDM were counterstained with Hoechst 33342 (Life Technologies, H3570, Darmstadt, Germany). Finally, 10 μl of the solution containing the parasites or BMDM were added to slides and covered with cover slips for microscopic examinations. Images were obtained using live video fluorescence and confocal microscopy systems (Wetzlar, Germany).

### Electrophoretic mobility shift assay (EMSA)

Genomic DNA was isolated from 1.5 × 10^9^ *L*. *major* promastigotes using Purelink Genomic DNA kit (K1820-01, Life Technologies, Germany) and was quantified by using a NanoDrop 2000 spectrophotometer (Thermo Scientific, Germany**)**. PHMB/gDNA polyplex formulations were prepared by adding and mixing 4.5 μg of gDNA with PHMB (0–54 μg) in a final volume of 30 μl water. Similarly, PHMB/CPG ODN or PHMB/CpG-R polyplexes were prepared by mixing 30 μg CpG ODN 1668 (M_w_ = 6058 g/mol, sequence TCCATGACGTTCCTGATGCT, Eurofins MWG Operon, Germany) or 15 μg of CpG-R with PHMB (0–54 μg) in a final volume of 30 μl water, respectively. After thorough mixing by pipette, the polyplexes were left at room temperature for 2 h prior to analysis. The samples were then combined with 6× Blue/Orange loading dye (Promega, Germany) and loaded onto a 1% agarose gel containing SYBR gold nucleic acid gel stain (S11494, Life Technologies, Germany) for electrophoresis.

### Transmission electron microscopy

Transmission electron microscopy (TEM) was used to determine particle sizes, and characterize stability and morphology of PHMB/CPG ODN polyplexes. For stability testing, polyplexes were prepared by mixing PHMB and CpG ODN at a 2:1 (w/w) ratio and kept at 4°C for two months. The samples were then mounted on 300-mesh grids, stained with uranyl acetate and lead citrate, analyzed with TEM (JEOL JEM-2100, Germany) operated at 80 kV and 200 kV with TVIPS F416 4k x 4k and Olympus Veleta 2k x 2k camera systems and imaged at 4400 or 12,000 x magnification.

### Dynamic light scattering (DLS) and electrophoretic light scattering (ELS)

The particle sizes and zeta potential of PHMB/CpG ODN polyplexes were measured by using a Beckman coulter Delsa Nano C (Beckman Coulter, Krefeld, Germany). The CONTIN analysis mode and the Smoluchowski equation were used to determine the size and the Zeta potential, respectively. The measurements were done in triplicate and reproduced at least twice. The size and zeta potential measurements were performed in clean disposable cuvettes containing 500 μl Millipore Water or ionic strength adjusted (ISA) water (KCl 0.15M) and PHMB/CpG ODN polyplexes at a ratio of 2:1 or 1:1 (w/w). The average particle size distributions of the polyplexes with their polydispersity index (PDI) and zeta potential values were determined by DLS and ELS. All measurements were carried out at 25°C. Scattered light was detected in an angle of 165° (particle size) and 15° (zeta potential).

### Isothermal titration calorimetry (ITC)

Affinity and specificity of interactions between PHMB and CpG ODN were studied by directly measuring the heat released or absorbed during binding events with MicroCal iTC200 System (GE Healthcare Northampton, MA, USA). All the samples were diluted in Millipore Water and degassed by ultrasonication for 15 min prior to their use. A 40-μl microsyringe was filled with 0.3 mM PHMB and injected at a rate of 2 μl/injections into cell samples containing 200 μl of 0.03 mM CpG ODN for a total of 20 individual injections. The titrant syringe functions as the stirrer at a speed of 400 rpm. The stabilization delay of the heat signal before the first injection was 3 min. The injection interval was 150 seconds and a calibration power of 5 ± 0.5 μcal/sec was used. The measurements were performed in triplicate at 25°C. The background heat was determined by titrating Millipore Water into CpG ODN and was subtracted from the main experiment. All data were automatically collected and analyzed using Origin and SigmaPlot Software to determine the thermodynamic parameters. The binding isotherm (*ΔH*) was used for fitting to an appropriate model.

### Determination of cytokine productions

BMDM were generated from female BALB/c mice as described above in cell culture. 1 × 10^6^ BMDM were seeded in a final volume of 0.5 ml in 24-well plates, infected with *L*. *major* promastigotes at 15:1 parasite to BMDM ratio, washed and incubated at 37°C for 30 min in complete RPMI medium containing CpG ODN (15 μg/ml). PHMB, CpG ODN or PHMB/CpG ODN polyplexes were added to the pre-activated or control cells and further incubated for 48 h. The supernatants were collected and IL-6, IL-10, IL-4 or TNF-α (Biosciences, Wiesbaden, Germany) concentrations were measured by sandwich ELISA while IL-12p70 was measured by using the IL-12p70 ELISA Ready-SET-Go kit from eBioscience according to the suppliers' instructions.

### Statistical analysis

Normalization and percentage calculations were based on mean fluorescence intensity (MFI) of the treated cells as compared to the control in flow cytometry data analyses. The values are given as mean ± standard error (SE). Statistical significance was determined by one sample t-test (Microsoft Excel Software).

### Ethics statement

All mice used in this experiment were kept under specific pathogen-free conditions and were handled in strict accordance with the German Animal Welfare Act 2006 (TierSchG).The animal protocol was approved by the government of Lower Franconia, Germany with permission number: 55.2–2531.01-26/12.

## Results

### Antileishmanial effects of PHMB and its proposed mechanisms of action

**Fig 1:**
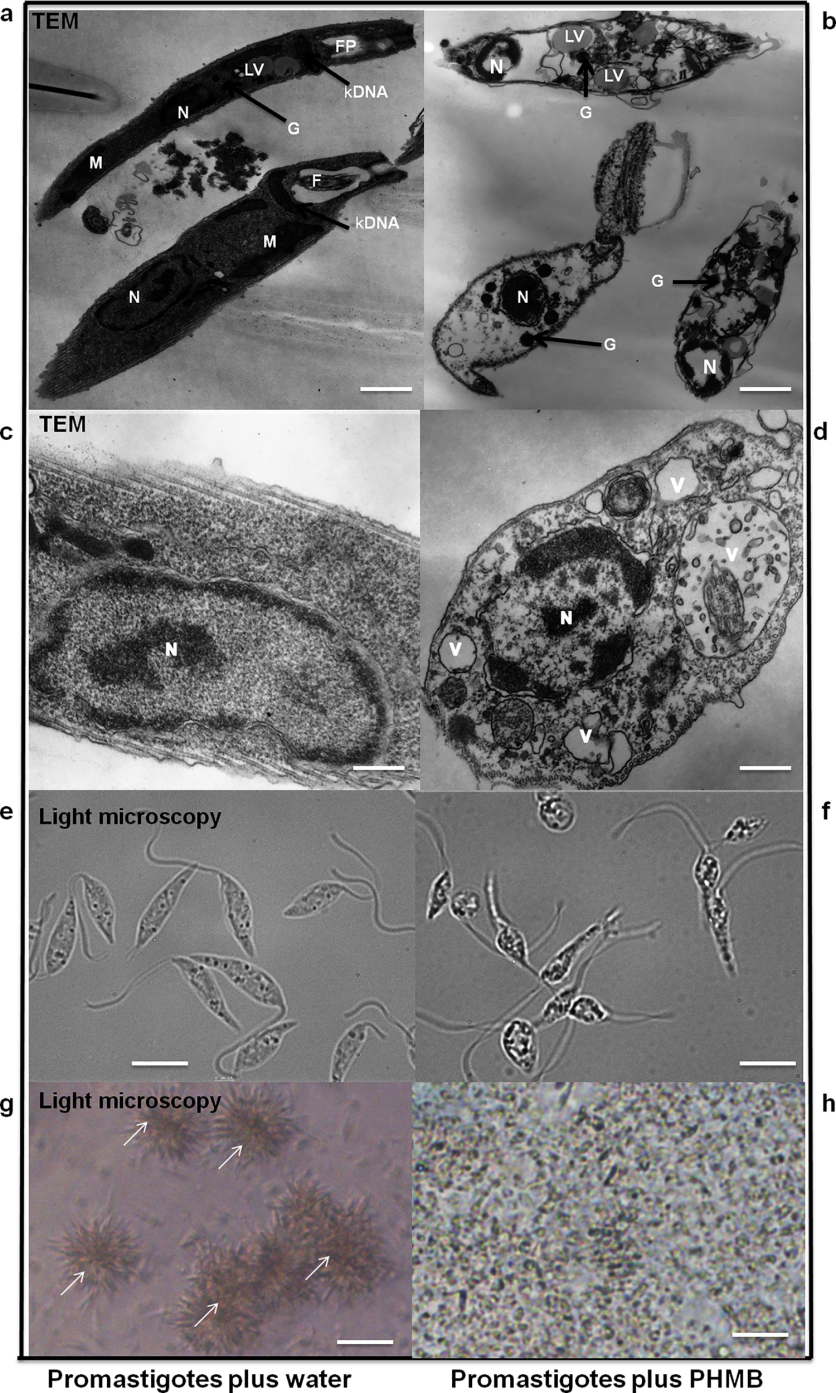
Effects of PHMB on *L*. *major* morphology and behavior. (b and d) TEM images of *L*. *major* promastigotes (right) showing morphological changes such as shrinkage, extensive cytoplasmic vacuolization (V), marked loss of cytosolic contents and condensed nucleus (N). Images b and d were taken after treatment with 2 μM PHMB for 48 and 24 h, respectively. (a and c) The untreated controls (left) show normal elongated morphology of promastigotes with intact and clear distinct kinetoplast (kDNA), N, mitochondrion (M), lipid vacuoles (LV) and glycosome (G). (e-h) Light microscopy images showing morphological and behavioral changes (no more clamp formation as indicated by arrowheads) after treatment with 2 μM PHMB for 24 h. Scale bars = (a) 1.1 μm, (b) 0.6 μm, (c and d) 0.25 μm, (e and f) 7.5 μm and (g and h) 25 μm.

To investigate PHMB’s potential use as a topical therapeutic agent against CL, its antileishmanial activity against the extracellular form of *L*. *major* (promastigotes) was assessed using an AlamarBlue cell viability assay [33]. PHMB effectively killed the promastigotes at sub-micromolar concentrations in a dose-dependent manner (S2 Fig). Determination of 50% inhibitory concentrations (IC_50_ values) confirmed PHMB’s activity (IC_50_ = 0.41 μM) against *L*. *major* promastigotes *in vitro*, with higher potency than the current standard antileishmanial drugs used in clinics (Table 1). PHMB was found to be 39-fold more potent than pentamidine, 69-fold more potent than miltefosine and 1,000-fold more potent than paromomycin, the currently recommended drug for treatment of CL. Visual examinations by light microscopy and TEM clearly revealed morphological and behavioral changes to promastigotes following treatment with PHMB (Fig 1). Furthermore, we investigated the bioactivity of PHMB against the intracellular form of the parasite (amastigotes) in *Leishmania-*infected macrophages model. The IC_50_ value of PHMB against intracellular amastigotes was 4 μM, suggesting that the potency of PHMB is lower against amastigotes relative to promastigotes.

**Table 1:** Antileishmanial and cytotoxic effects of PHMB and PHMB/DNA polyplexes. The tables show antileishmanial efficacy, cytotoxicity and selectivity of the compounds. The IC_50_ values and/or selectivity index (SI) of the compounds as compared to standard antileishmanial drugs are shown. The table values are the average IC_50_ values of 3–6 independent experiments for each compound. IC_50_ = minimum concentration of the substances required to kill 50% of the population; SI = mean IC_50_ against BMDM /mean IC_50_ against *L*. *major* amastigotes; ND = not determined.

Substances	IC_50_ (μM)	IC_50_ (μM)	IC_50_ (μM)	SI
	promastigotes	BMDMs	amastigotes	amastigotes
PHMB	0.41 ± 0.25	4± 0.7	4 ± 0.65	1
CpG ODN	> 150	>150	>150	ND
PHMB/CpG (1:2 w/w)	2.54 ± 0.5	134 ± 40.2	1.12 ± 0.23	119.4
PHMB/CpG (1:1 w/w)	2.2 ± 0.28	50 ± 10.4	2. 82 ± 2.8	17.7
PHMB/CpG (2:1 w/w)	1.45± 0.33	33 ± 9.5	1± 0.55	15.89
Paromomycin	>500	>900	1500 ± 112.5	ND
Miltefosine	28.33 ± 5.2	75.6 ± 10.9	54 ± 8.1	1.4
Pentamidine	16.19 ± 3.1	127.6 ± 24	6.38 ± 2.4	20
Amphotericin B	0.42 ± 0.5	> 21	0.099 ± 0.05	>208

To determine the level of pathogen/host selectivity of PHMB, we measured its cytotoxicity against primary bone marrow-derived macrophages (BMDM), keratinocytes and 293T epithelial cells using an AlamarBlue assay. The IC_50_ values obtained indicate that PHMB is about 6.5 fold more toxic against BMDM (IC_50_ = 4 μM) than 293T epithelial cells (IC_50_ = 26 μM). Moreover, PHMB killed 50% of keratinocytes at 6.6 ± 3.5 μM. The results show that keratinocytes are more sensitive to PHMB relative to 293T epithelial cells but less sensitive than BMDM. Although PHMB is currently being safely used for several purposes in clinics and industries, our results showed that PHMB has poor selectivity between amastigotes and BMDM with a selectivity index of 1 (Table 1). Of course, it is important to consider that these values are for cells grown in culture.

**Fig 2:**
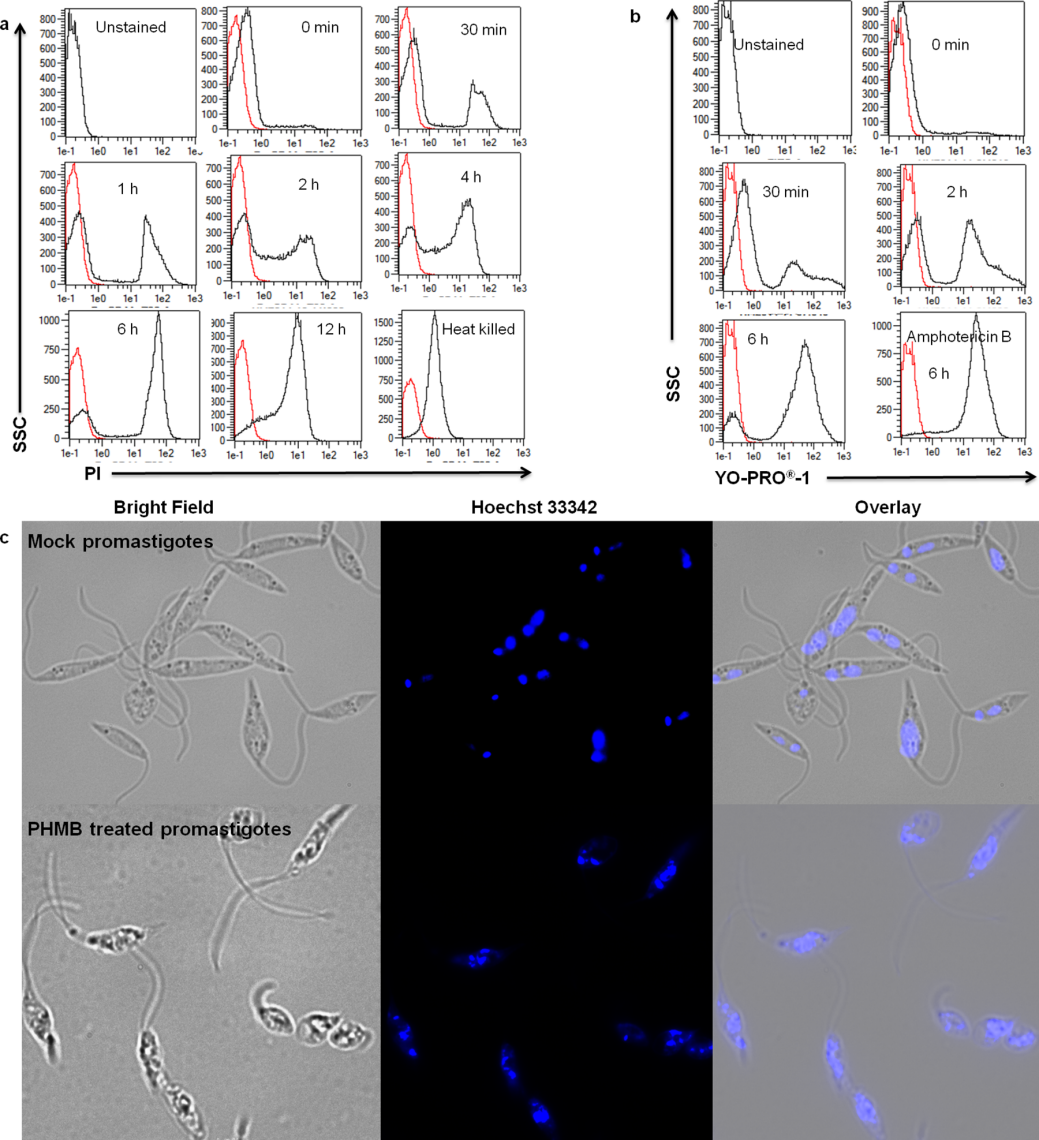
Mechanism(s) of action of PHMB on *L*. *major* promastigotes. PHMB disrupted membrane integrity and condensed DNA in *L*. *major* promastigotes. Representative FACS histogram (a) propidium iodide (PI) and (b) YO-PRO-1dye staining of promastigotes after treatment with 2 μM PHMB for indicated time points, showing time-dependent effect of PHMB on the membrane integrity. Heat-killed promastigotes and amphotericin B at 2 μM concentration were used as positive controls. (c) Fluorescent microscopy analysis showing condensed and damaged DNA materials of *L*. *major* promastigotes after treatment with PHMB at 2 μM for 48 h as compared to the mock control (distilled water). Scale bars = 5 μm.

Encouraged by the potency of PHMB against *L*. *major* promastigotes, we investigated its underlying mechanism(s) of action. Literature mining suggested that PHMB’s microbe-selective toxicity is due to preferential disruption of microbial membranes over mammalian membranes [38,39]; it is believed to specifically interact with acidic phospholipids of microbial pathogens, whereas the neutral phospholipids in mammalian cell membranes are only marginally affected [38,40]. We hypothesized that PHMB also kills *L*. *major* parasites by this mechanism, and tested the effects of PHMB on the membrane integrity of the promastigotes by using propidium iodide (PI), a dye that can enter the parasite and stain DNA only after loss of membrane integrity. After incubating promastigotes with 2 μM PHMB for 30 min, more than 20% of the parasite population was stained by PI and staining increased at later time points (Fig 2A). We repeated the dye exclusion assay using YO-PRO-1 dye according to the manufacturer’s instructions, and the results showed similar time-dependent permeabilization (Fig 2B). Therefore, PHMB damaged the membrane integrity of parasites in a time-dependent manner and permeabilized parasites were observed within 30 minutes, supporting the hypothesis that PHMB disrupts parasite membranes.

**Fig 3:**
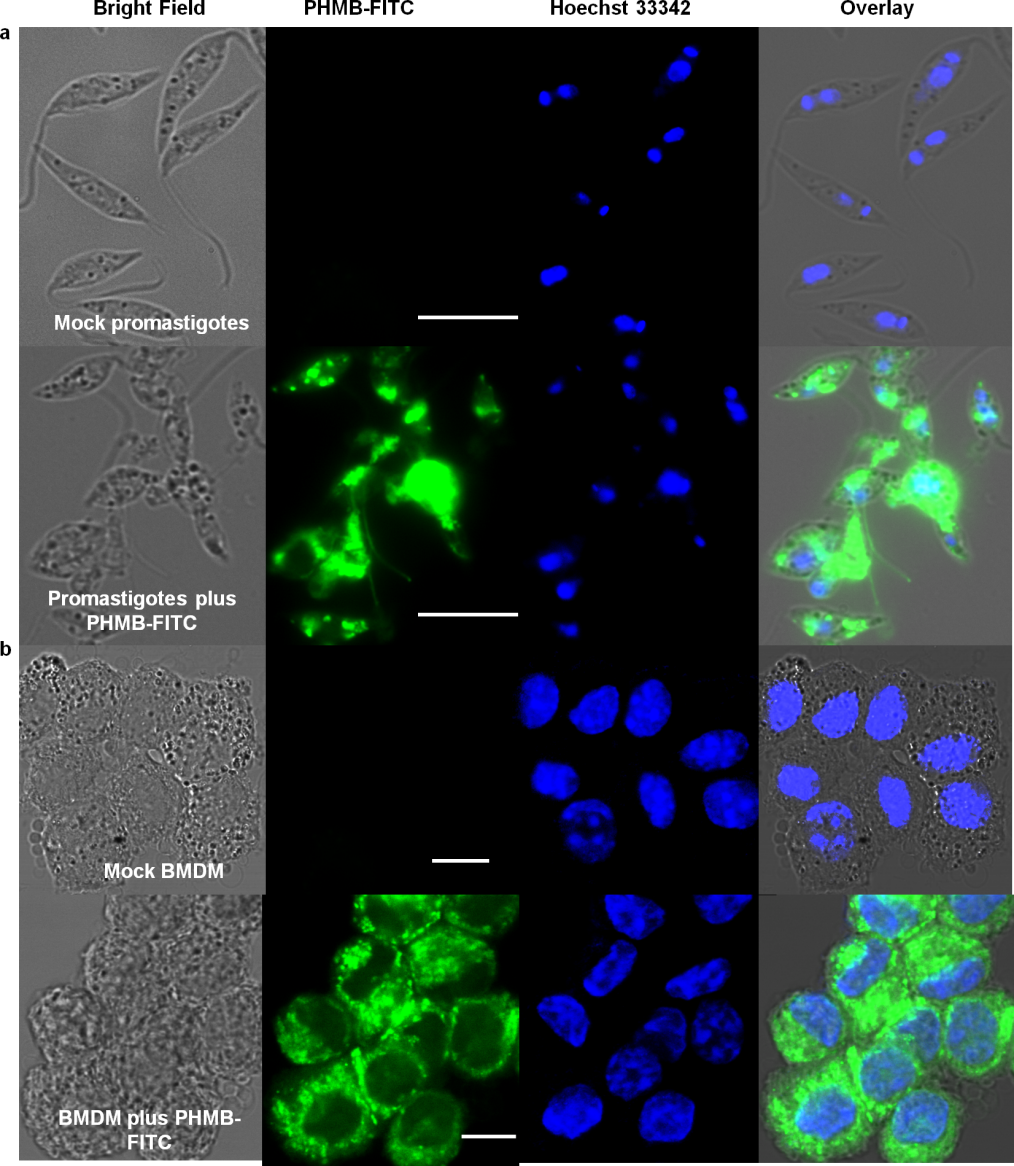
Cell localization of PHMB-FITC in promastigotes and BMDM. Confocal images showing uptake of PHMB-FITC by (a) promastagotes and (b) BMDM. Note apparent nuclear exclusion in BMDM cells but not parasites, scale bars = 10 μm.

**Fig 4:**
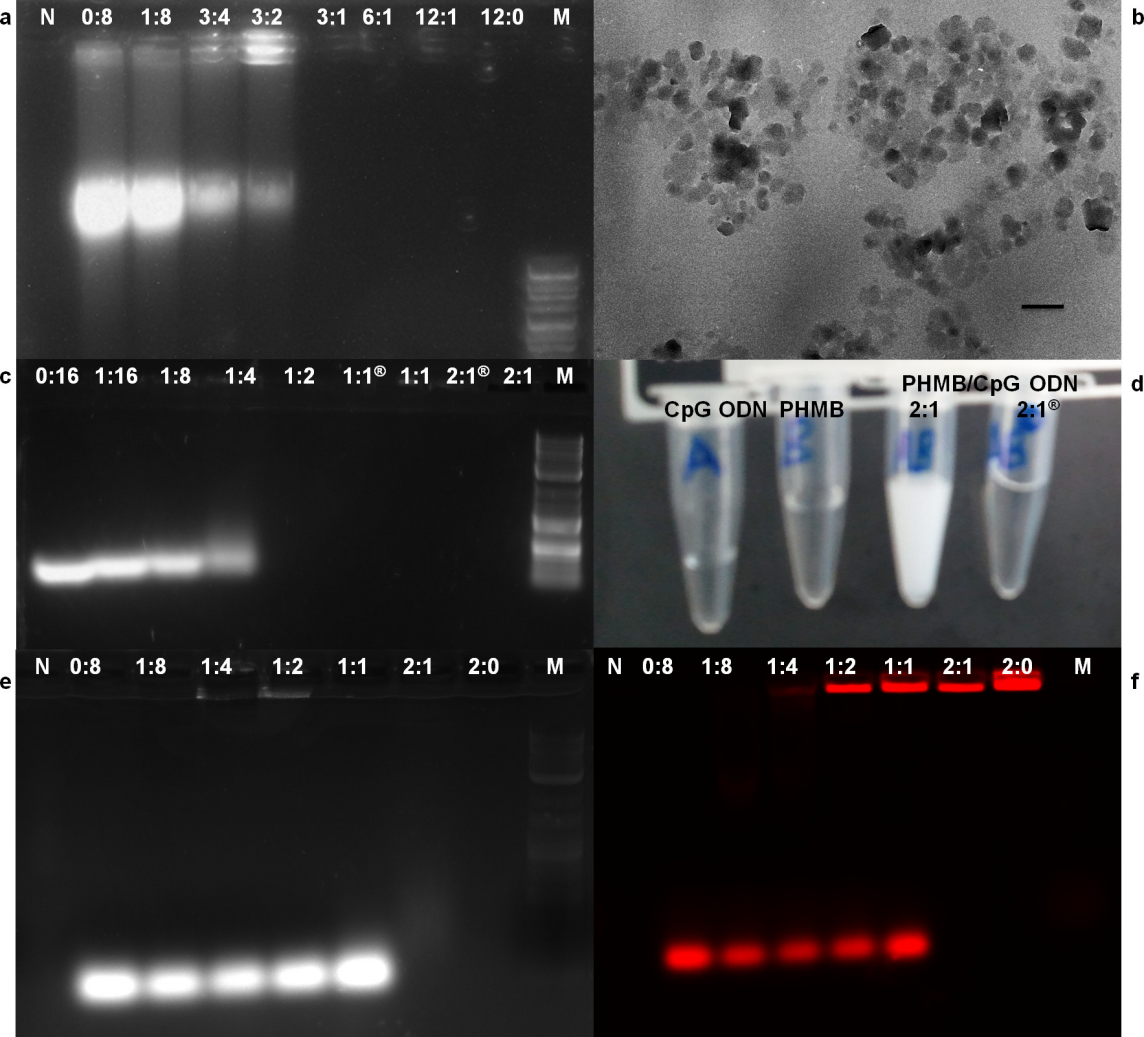
PHMB/DNA interactions and physicochemical characterization of polyplexes. Formation of PHMB/DNA polyplexes confirmed by 1% agarose gel, TEM and color change. (a) PHMB/gDNA polyplexes, (b) TEM picture showing PHMB/CpG ODN polyplexes formation at 2:1 ratio, (c) PHMB/CpG ODN polyplexes, (d) temporary turbidity change during PHMB/CpG ODN complexation, (e) PHMB/CpG-R polyplexes and (f) the same gel (e) with fluorescence measurement of CpG-R. With the exceptions of negative control and PHMB alone, all wells contained equal amounts (weight) of DNA with various concentrations of PHMB. M represents 2-Log DNA ladder (0.1–10.0 kb, New England Biolabs) and N represents water used as a negative control. All indicated PHMB/DNA ratios are in relative weight (w/w). Indicates two month old PHMB/CpG ODN polyplexes. Scale bar = 300 nm.

During our TEM ultrastructure analysis, we consistently observed condensation of parasite nuclei after incubation with 2 μM PHMB for 24─48 h (Fig 1). Moreover, PHMB’s mechanism of bactericidal action has been associated with its strong and cooperative interaction with nucleic acids *in vitro* [41]. This led us to consider that PHMB’s parasite killing mechanism(s) may also involve binding to DNA inside parasites. To test this possibility, promastigotes were treated with 2 μM PHMB for 48 h, stained with Hoechst 33342 and examined by confocal and fluorescence microscopy. The results indicated that PHMB bound, condensed and disrupted chromosome structures (Fig 2C). This interpretation of the cellular effects would require polymer entry into parasites and also strong PHMB binding to parasite genomic DNA (gDNA). To test parasite entry, *L*. *major* promastigotes were incubated with a derivative of PHMB that was covalently tagged with fluorescein isothiocyanate (FITC) at 0.35 μM (sub-IC_50_). The results confirm that parasites take-up PHMB-FITC, and the polymer distributes into nuclei, where it could interact with chromosomes (Fig 3A). To test chromosome binding, we used isolated genomic DNA (gDNA) from *L*. *major* and an electrophoretic mobility shift assay (EMSA). Clear migration retardation confirmed that PHMB and gDNA form polyplexes at relative weight ratios ≥ 0.25, and, thus, may also bind chromosomal DNA within parasites (Fig 4A).

We next considered how PHMB selectively condenses or disrupts parasite chromosomes without affecting host DNA. To investigate the cellular localization(s) of PHMB, we used its fluorescent version (PHMB-FITC). BMDM cells were incubated with 1 μM PHMB-FITC, counter stained with Hoechst 33342, and internalization was investigated using fluorescence and confocal laser scanning microscopy. The data show that PHMB readily enters BMDMs but localizes in the cytoplasm without apparent entry into nuclei in mammalian cells, indicating failure to transit across the nuclear envelope (Fig 3B). Therefore, intracellular localization of PHMB appears to differ in BMDMs and parasites, and PHMB gains access to parasite chromosomes but not host macrophage chromosomes.

### PHMB is a novel non-viral carrier of CpG oligonucleotides

Two major observations encouraged us to hypothesize that PHMB in combination with immunomodulatory nucleic acids could be used as parallel host- and pathogen-directed killing of an intracellular *L*. *major*. Primarily, we knew that it binds genomic DNA of *L*. *major* parasites forming PHMB/gDNA complexes. Moreover, PHMB has been used as an siRNA transfection vehicle in hybrid form with iron oxide [42]. To test whether nucleic acid delivery could be improved using PHMB, CpG ODN was selected as a model immunomodulatory nucleic acid molecule. CpG ODN is a potent immune modulator that activates macrophages and dendritic cells to kill intracellular *L*. *major* parasites [43]. We reasoned that combinations of PHMB and CpG ODN could potentially kill parasites directly through parasite membrane and chromosome disruption and indirectly through modulation of host immunity. If successful, such dual host- and pathogen-directed effects could improve potency. In this context, a range of PHMB/CpG ODN polyplex formulations were prepared and characterized, and their bioactivities were evaluated and compared to the free components.

Before evaluating the potential use of PHMB as a safe and effective carrier for nucleic acids, we first wanted to test whether PHMB binds CpG ODN to form stable nanocomplexes. TEM results showed that there is complex formation between PHMB and CpG ODN at 2:1 ratio (Fig 4B). Our EMSA experiments showed that PHMB retards CpG ODN mobility at relative weight ratios ≥ 0.25 (Fig 4C). Following addition of an excess of PHMB to CpG ODN, we also observed an instant reaction that converted colorless PHMB and CpG ODN in solution to a temporal milk-like appearance, suggesting electrostatic interactions between PHMB and CpG ODN and complex formation (Fig 4D). Consistent with complex formation, the ODN in the presence of PHMB showed migration retardation when analyzed by an EMSA. To more clearly visualize oligo mobility changes, the same CpG ODN was labeled with rhodamine red at 3’ termini (CpG-R), and the results confirmed retarded electrophoretic mobility and retention of CpG ODN in the wells (Fig 4E and 4F).

**Fig 5:**
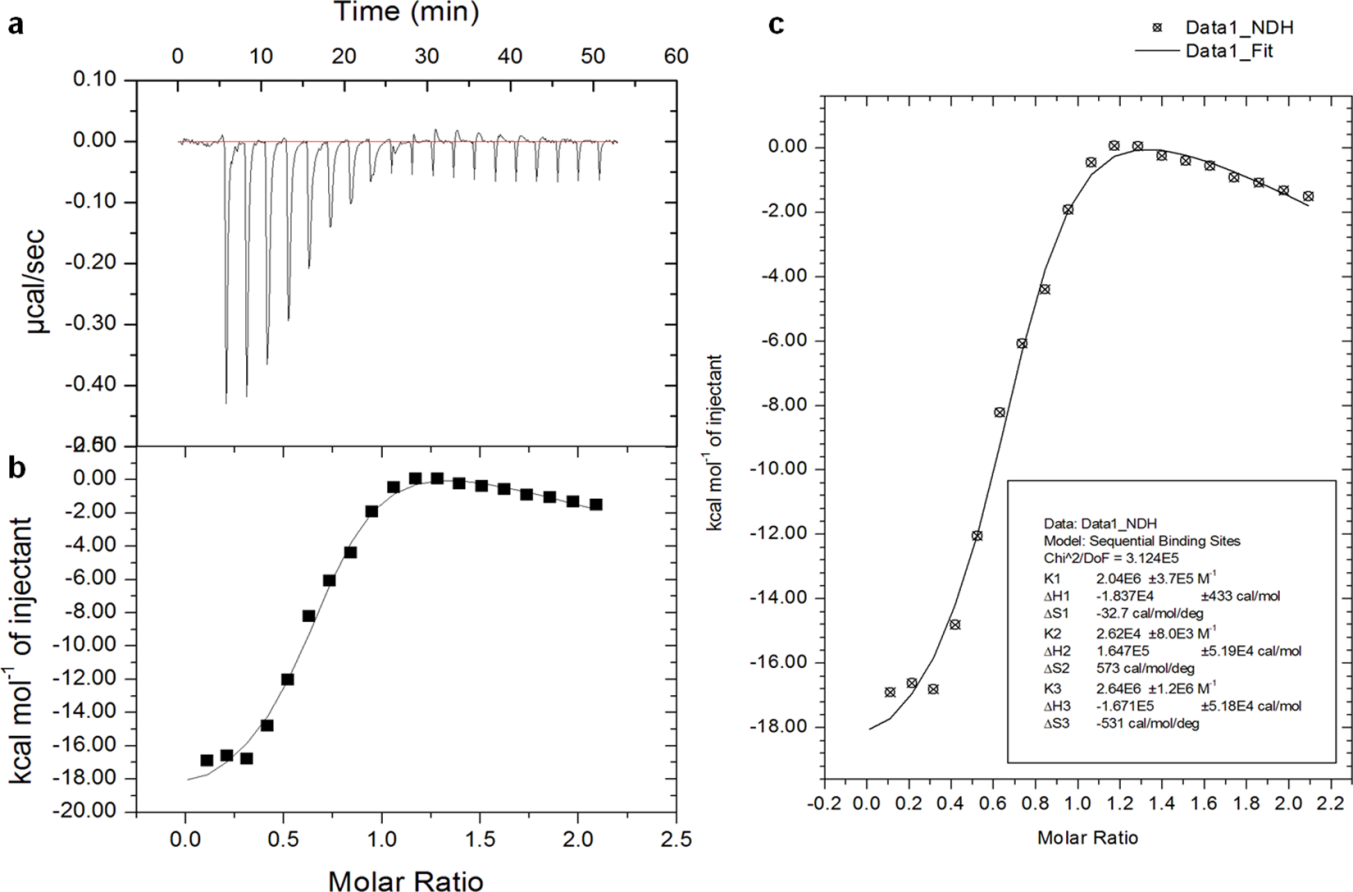
Physicochemical characterization of PHMB/CpG ODN nanopolyplexes. The figures show quantitative measurements of the strength of interaction between CpG ODN and PHMB by ITC with the corresponding thermodynamic parameters: binding affinity (*K*_a_), enthalpy changes (Δ*H*) and entropy changes (Δ*S*). (a) Raw data generated by ITC, (b) and (c) analyzed ITC data using two data analysis and plotting tools: Origin and SigmaPlot, respectively.

We next characterized the size and surface charge of complexes using DLS and ELS. These analyses indicated particles of highly reproducible sizes: 310.9 ± 30.5 nm and 285.3 ± 15.0 nm at relative weight ratios of 2:1 and 1:1, respectively (Table 2). The PDI values of the polyplexes were less than 0.3, indicating a narrow size distribution. To assess the stability of the polyplexes, their zeta potential was measured by ELS and was found to be + 33.3 ± 1.1 mV (ratio 2:1) and—18.77 ± 0.4 mV (ratio 1:1), and dependant on the chosen ratio of PHMB and CpG ODN (Table 3). As expected, higher PHMB to CpG ODN ratios resulted in polyplexes with higher positive relative zeta potentials. The data show that PHMB/CpG ODN complexes are moderate stable at 2:1 ratio, whereas they may not be stable at a 1:1 ratio. To evaluate stability over time, PHMB/CpG ODN poylplexes were prepared and kept at 4^°^C for two months before their physicochemical nature were again characterized by TEM and EMSA. Similar results were obtained and, thus, confirmed the stability of the polyplexes over time at 2:1 ratio (Fig 4B and 4C indicated by ®). To quantify the strength and specificity of the interactions between the two molecules, we used isothermal titration calorimetry (ITC). The results show that PHMB can strongly interact with CpG ODN to form polyplexes, confirming its binding affinity towards DNA. The detailed thermodynamic parameters indicate sequential binding between PHMB and CpG ODN (Fig 5). The ITC data show that the reaction enthalpy of the PHMB/CpG ODN binding was initially exothermic (-18.37 kcal/mol), indicating a strong electrostatic interaction between the negatively charged phosphate backbone of CpG ODN and the cationic charged polymer PHMB. The exothermic signals declined during the course of titration and were found to plateau at molar ratio of 1:1 of both binding partners. Finally, the TEM results also confirm nanopolyplex formation between PHMB and CpG ODN, and further suggest a predominantly spherical morphology for the polyplexes, rather than cylindrical or rod forms (Fig 4B). Taken together, the results confirmed that there is strong electrostatic interaction between PHMB and CpG ODN that leads to formation of moderate stable polyplex nanoparticles.

**Table 2:** Dynasore rescues killing of *L*. *major* amastigotes inside macrophages. Dynasore inhibited killing of the intracellular *L*. *major* amastigotes and promastigotes by PHMB or PHMB/CpG ODN polyplexes. Values are the average of two independent experiments and the values are given as mean ± SE. The concentration values given are for PHMB within polyplexes.

Substances	IC_50_ (μM) amastigotes	IC_50_ amastigotes + dynasore (μM)	IC_50_ (μM) promastigotes	IC_50_ promastigotes + dynasore (μM)
PHMB	4 ± 0.7	8.1 ± 2	0.41 ± 0.25	0.697 ± 0.85
PHMB/CpG (1:2)	1.12 ± 0.23	>14.2	2.54 ± 0.5	4.8 ± 0.94
PHMB/CpG (1:1)	2.82 ± 2.8	>14.2	2. 2 ± 0.28	3.93 ± 0.82
PHMB/CpG (2:1)	1 ± 0.6	>14.2	1.45 ± 0.33	2.61 ± 0.41
Dynasore (80 μM)	No activity	No activity	No activity	No activity

**Table 3:** Physicochemical characterization of PHMB/CpG ODN nanopolyplexes. The table shows particle size and zeta potential determination by using DLS and ELS with their PDI. The table summarizes the results of three independent experiments. Size distribution and zeta potential measurements were performed using Millipore water and ISA water (0.15M KCl), respectively.

Ratio of PHMB/ CpG ODN (w/w)	Particle size (nm)	Polydispersity index (PDI)	Zeta potential (mV)
2:1	310.9 ± 30.5	0.222 ± 0.04	+ 33.3 ± 1.1
1:1	285.3 ± 15.0	0.260 ± 0.02	- 18.77 ± 0.4

**Fig 6:**
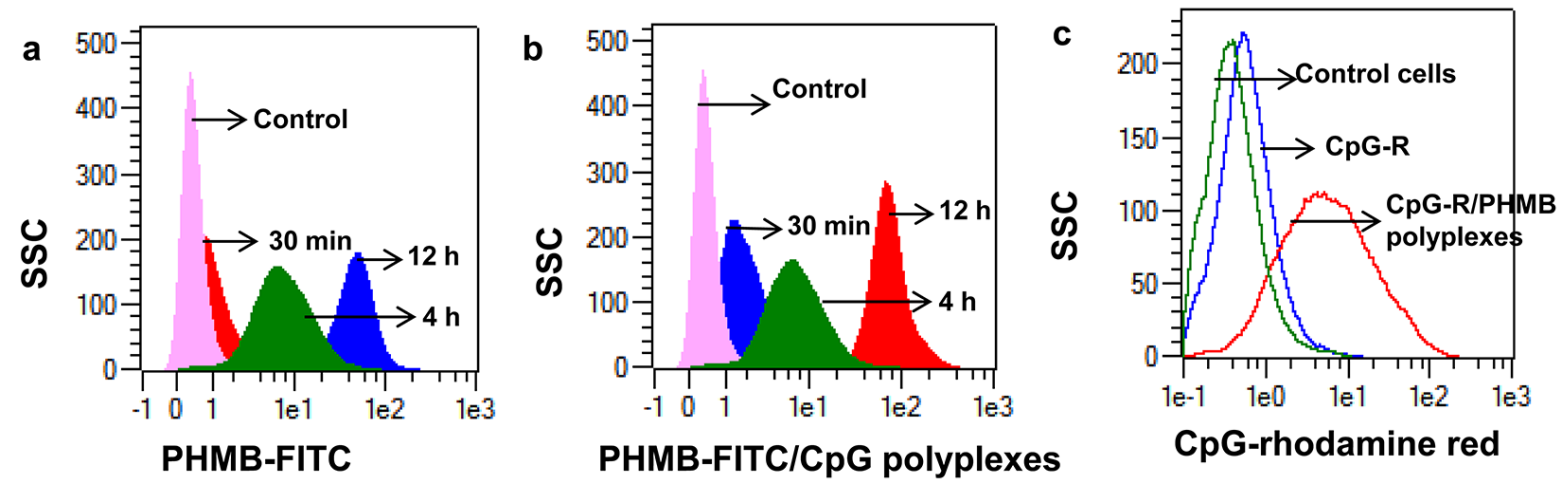
Cellular uptake of PHMB and CpG ODN, and their polyplxes by BMDM. The overlay histograms show time dependent uptake of (a) PHMB-FITC and (b) PHMB-FITC/CpG ODN polyplexes into macrophages. Whereas (c) shows uptake of CpG-R by BMDM as polyplex form compared to its free form. The histograms are representative of three independent experiments.

**Fig 7:**
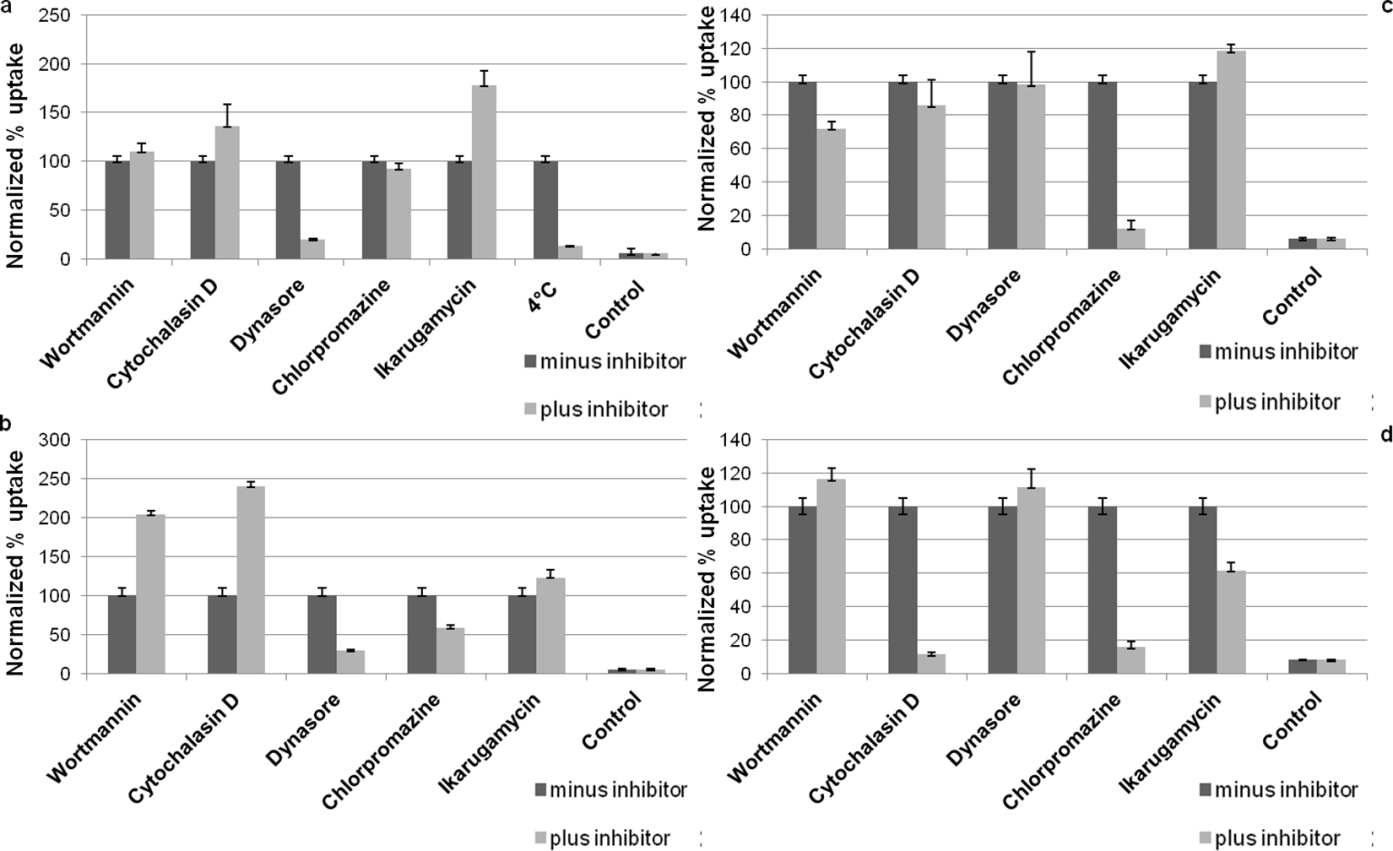
Cellular uptake of PHMB-FITC and PHMB-FITC/CpG ODN polyplexes and effects of selective endocytosis inhibitors. The bar graphs show the effects of different inhibitors and temperature on cellular uptake of (a) PHMB-FITC, (b) PHMB-FITC/CpG ODN polyplexes, (c) dextran/FITC used as positive control to exclude the effect of FITC that might interfere with the cellular uptake mechanisms of PHMB and (d) alexa-448-labeled transferrin used as positive control for clathrin-dependent endocytosis. Normalized MFI values of three independent flow cytometry experiments are shown and the values are given as mean ± SE.

We next tested the potential to use PHMB as a safe and effective delivery vehicle for CpG ODN. We characterized the polyplexes by measuring uptake efficiency, cytotoxicity, anti-parasitic efficacy and their immune modulating activity relative to the free components. The uptake efficiency of CpG-R in the polyplex form was enhanced about 15-fold relative to the free form, while the uptake of PHMB as a polyplex was not enhanced, confirming that PHMB can deliver DNA molecules into macrophages (Fig 6). Because PHMB and CpG incorporation into polyplex condensates may quench the mean fluorescence of PHMB-FITC or CpG-R, these uptake measurements may underestimate delivery. To learn more about the mechanism of entry, we investigated their uptake properties into macrophages. Confocal microscopy and flow cytometry results showed that PHMB-FITC is readily taken up by macrophages in a time-dependent manner (Figs 3B and 6A). In contrast, when the PHMB-FITC was incubated with BMDM at 4°C under equivalent cell culture conditions, uptake was strongly reduced (87%) (Fig 7A), indicating that the predominant uptake mechanism of PHMB-FITC by BMDM is *via* an energy-dependent mode, which is likely to be endocytosis. To identify the specific endocytosis pathway(s) involved, a wide range of selective endocytosis inhibitors were employed [32]. Briefly: we used: 1) wortmannin, a potent inhibitor of phosphatidylinositol kinase (PI3K) that is commonly used as inhibitor of macropinocytosis, 2) cytochalasin D, which blocks the actin polymerization and is commonly used as an inhibitor of phagocytosis, 3) dynasore, an inhibitor of dynamin-dependent endocytosis, 4) chlorpromazine hydrochloride, which inhibits a Rho GTPase that is essential for the formation of clathrin-coated vesicles in clathrin-dependent endocytosis, and 5) ikarugamycin, an inhibitor of clathrin coated pit-mediated endocytosis. Cells were pre-incubated with specific endocytosis inhibitors for 30 min before exposure to PHMB-FITC, the polyplexes, dextran or transferrin, followed by either incubation at 4°C or 37°C for 4 h. The results show that only dynasore, a cell-permeable small molecule that inhibits dynamin GTPase activity needed for dynamin-dependent endocytosis, effectively inhibited the uptake of PHMB-FITC (80% inhibition) (Fig 7A). Similar results were obtained for PHMB-FITC/CpG ODN and PHMB/CpG-R polyplexes (Figs 7B and S3), suggesting that the uptake pathways of PHMB by BMDM is not affected by a processes of polyplex formation. However, dextran and transferrin were effectively inhibited by cytochalasin D and chlorpromazine hydrochloride but not by dynasore. Taken together, the results show that the uptake mechanisms of PHMB-FITC and its polyplexes were predominantly *via* dynamin-dependent endocytosis.

We next assessed cytotoxicity against the host cells. Surprisingly, the results showed that IC_50_ values of the polyplexes were much higher (50 ± 10.4 4 μM and 33 ± 9.54 μM at 1:1 and 2:1 ratios, respectively) against BMDMs than the PHMB alone. Similarly, the toxicity of PHMB/CpG ODN polyplexes showed much higher IC_50_ values (185 ± 22.5 and 90.3 ± 23.5 at 1:1 and 2:1 ratio, respectively) against 293T epithelial cells than PHMB alone. The results indicate that the polyplexes have drastically reduced toxicity against host cells as compared to free PHMB. Similarly, we checked the cytotoxicity of the polyplexes against *L*. *major* promastigotes and the results show lower efficacy as compared to free PHMB, and CpG ODN alone did not show activity (Table 1). The cationic nature of polymers (higher positive zeta potential) is often associated with toxicity against host cells and decationizing is expected to reduce toxicity [44]. As expected, the toxicities of the polyplexes against BMDM and 293T cells increased as the relative weight ratio of PHMB:CpG ODN was increased (Table 1). These results are consistent with our zeta potential data showing that increasing PHMB/CpG ODN ratios increased polyplex positive charge (Table 3).

We then determined leishmanicidal effects of PHMB and CpG ODN in polyplex and free forms using BMDM infected with the luciferase-transgenic *L*. *major* strain. The polyplexes displayed enhanced antileishmanial activity against intracellular amastigotes while increasing selectivity many fold (Table 1). We hypothesized that enhanced antileishmanial activity was due to increased host cell delivery. If this was the case, blockage of the uptake pathway using dynasore would suppress PHMB’s intracellular killing of amastigotes. Accordingly, we determined the efficacy of PHMB or its polyplexes against intracellular amastigotes in the presence/absence of dynasore. The results show clearly that dynasore inhibits PHMB-mediated killing of amastigotes (Table 2). In the presence of dynasore (80 μM), the IC_50_ value of PHMB against the intracellular amastigotes was increased by two fold and the polyplexes were completely inactive until the maximum dose tested (14.2 μM) as compared to the polyplexes without dynasore (1–2.28 μM). Similarly, dynasore inhibited the antileishmanial effects of PHMB and the polyplexes against promastigotes. To learn more about the effect of dynasore on PHMB mediated killing of promastigotes, we investigated the mechanism by which the promastigotes take up PHMB. We used sub-IC_50_ doses of PHMB-FITC (0.35 μM) and the same inhibitors used earlier with BMDMs. Surprisingly, the results showed that dynasore inhibited the uptake of PHMB-FITC into promastigotes by more than 98% while other inhibitors did not significantly alter uptake (S4 Fig). Dynasore alone did not display cytotoxicity against either amastigotes or promastigotes (Table 2). Therefore, dynasore rescued the parasites from the intracellular antileishmanial effects of PHMB by blocking polymer uptake into macrophages, further confirming its intracellular killing potential, consistent with our evidence of a DNA-level mechanism of action. Moreover, the results suggest that dynasore also blocks the uptake of PHMB by promastigotes and, consequently reduced the antileishmanial effects. Taken together, the results suggest that PHMB is an effective antileishmanial compound and a nucleic acid entry-promoting vehicle for host- and pathogen-directed therapy.

**Fig 8:**
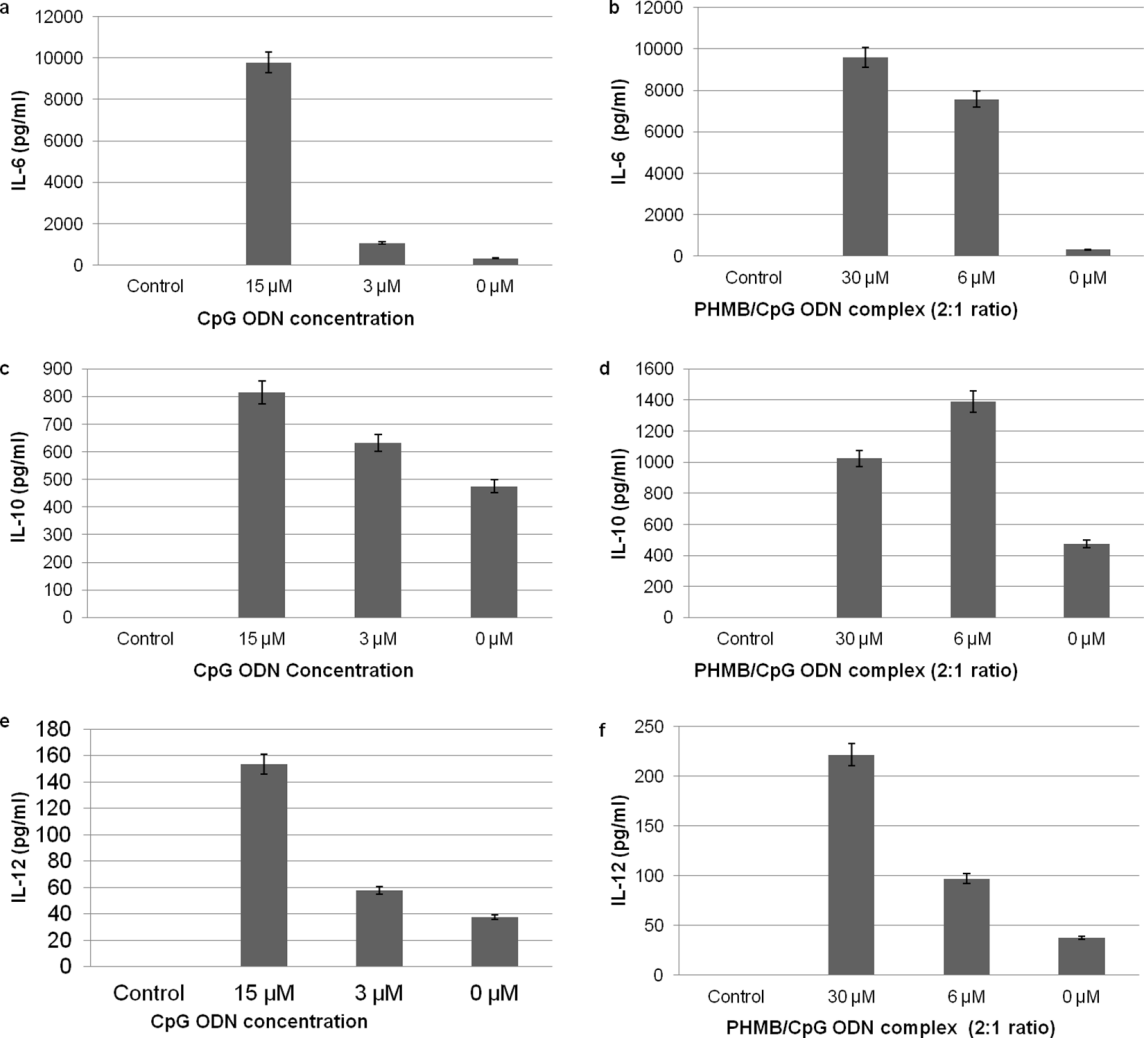
Effects of CpG ODN and PHMB/CpG ODN polyplexes on cytokine production by BMDM. The bar graphs show the levels of (a-b) IL-6, (c-d) IL-10 and (e-f) IL-12 production after CpG ODN or PHMB/CpG ODN polyplexes were added to BMDM that had been pre-activated with CpG ODN (15 μg/ml) for 30 min and further incubated for 48 h. Controls were cells without any pre-stimulation and the 0 μM doses were cells pre-stimulated by CpG ODN but without any further stimulation by CpG ODN or the complex. The concentrations shown for the polyplexes represent the dose of PHMB and the concentrations of CpG ODN are half of the indicated doses. The error bars show standard errors from three independent experiments.

Finally, to assess whether PHMB has immunomodulatory properties and whether polyplex formation affects the immunomodulatory properties of CpG ODN, the concentrations of several key cytokines were measured by enzyme-linked immunosorbent assays (ELISA) taken from supernatants of co-cultured BMDM. For free CpG ODN, production of all cytokines tested increased with increasing doses (Fig 8). We were not able to obtain reliable measures of IL-4 and TNF-α in cells treated with polyplexes or the free components (S2 Table). We also observed that PHMB alone stimulated the production of proinflammatory cytokines such as IL-6, IL-10 and IL-12 by activating macrophages (S5 Fig). Moreover, based on the IL-6, IL-12 and IL-10 measurements, the polyplexes generally showed higher immune stimulation capacity as compared to free CpG ODN. This suggests that the polyplex components are bioactive after delivery into BMDMs; in other words, PHMB must release the CpG ODN from the polyplexes inside BMDM.

## Discussion

Leishmaniasis therapy relies on only a few chemotherapeutic agents, all of which lack appropriate efficacy, safety and affordability. Many drugs used as antiparasitics were repositioned from other disease areas [14], and this strategy seems appropriate for leishmaniasis, where a very low cost of goods is required. Here we demonstrate that the long-established antimicrobial polymer PHMB is a potent antileishmanial candidate drug for topical applications against CL. PHMB is more potent than the current standard antileishmanial drugs *in vitro* and may provide additional benefits through wound healing, immune modulation and cellular delivery of complementary drugs.

PHMB is a very inexpensive compound (about $10-50/kg) that has been widely used in clinics, homes and industry as a disinfectant and antiseptic, with excellent tolerance for over 50 years worldwide [40,45–47]. It has a solid safety record and is widely considered to be useful in wound care [29,48]. As typical for cationic polymers, cell toxicity is observed at high concentrations. PHMB toxicity was found to be unexpectedly high against BMDM, but was greatly reduced when complexed with ODN, presumably due to charge complexation/shielding, while retaining antileishmanial effects (Table 1). Thus, PHMB may be repositioned and potentially used as an effective topical drug against CL, following appropriate clinical trials, and complexation with immunomodulatory CpG oligos may provide additional benefits.

Our results suggest that PHMB directly kills parasites via a dual mechanism involving both permeabilization of parasite membranes and selective condensation and disruption of parasite chromosomes. Recently, it has been shown that PHMB readily enters diverse bacterial species and kills through chromosomal condensation (Chindera et al., submitted). Similarly, our results reveal that PHMB condenses or damages parasite chromosomes, but does not enter macrophage nuclei. Thus, the finding helps to explain how PHMB kills parasites without similar damage to host cells.

Successful chemotherapy of infectious diseases caused by intracellular pathogens, including *L*. *major*, involves modulation of host immunity for host-directed treatments. It has been shown that the recommended antileishmanial drugs have immunomodulatory properties and exert their effect in part via host immunomodulation [49,50]. Thus, a combination therapy comprising components that can directly kill the pathogen as well as activate host immune systems are attractive. Nucleic acids such as CpG ODN have potential applications both as adjuvants and nonspecific immune modulators by triggering immunity against intracellular pathogens like *L*. *major* [43]. They also hold great promise for therapy against many diseases as mediators of RNA silencing and vaccination. However, medical applications are severely hindered by their instability in biological fluids and poor cellular uptake [51]. Also, an efficient and safe delivery of nucleic acid-based immune modulators is a key challenge. Complexing nucleic acids with clinically compatible polymers to form nanoparticles could overcome the delivery challenges by improving passage across physiological or cell barriers. In the context of host- and pathogen-directed therapy, we investigated the nucleic acid delivery potential of PHMB as well as the overall synergistic effect of a combined therapy comprising PHMB and CpG ODN complexes against CL. PHMB and CpG ODN form self-assembled nanostructures that are readily taken up by macrophages, increasing the uptake efficiency of nucleic acids and increasing the potency and selectivity of PHMB against the parasites. In principle, polyplex formulation could improve delivery and provide sustained release.

The potential antileishmanial effects of PHMB are threefold. First, it can accelerate wound closure, thus improving the cosmetic outcome of CL and preventing secondary bacterial and/or fungal infections which often delay wound healing in CL patients [52]. Second, it can directly kill lesion-causing parasites. Third, the antileishmanial effects of PHMB may be further facilitated by its ability to deliver immunomodulatory agents such as CpG ODN. The discovery of PHMB-nucleic acid interactions and formation of nanopolyplexes for efficient delivery into macrophages may have vast applications beyond the initial objectives of this study, including therapy for other intracellular pathogens or non-infectious diseases.

## Supporting Information

S1 Fig Structure of PHMB. (TIF)

S2 Fig Dose-dependent antileishmanial activity of PHMB against *L*. *major* promastigotes. The error bars show the standard error of three independent experiments.
(TIF)

S3 Fig CpG ODN delivery into macrophages using PHMB. The cellular uptake potential of (a) PHMB/CpG-R and (b) PHMB-FITC/CpG ODN polyplexes, and inhibition by dynasore. Free PHMB-FITC, PHMB-FITC/CpG ODN and PHMB/CpG-R polyplexes were efficiently blocked by dynasore. Based on their MFI, the uptake of CpG-R was enhanced by about 15 folds as polyplex form compared to its free form.
(TIF)

S4 Fig PHMB-FITC uptake into promastigotes and the effects of selective endocytosis inhibitors. The bar graphs show the effects of different inhibitors on blocking the uptake of PHMB-FITC by promastigotes. Normalized mean fluorescence intensity (MFI) values of three independent flow cytometry experiments are depicted as mean ± SE.
(TIF)

S5 Fig Effects of PHMB on cytokine production by BMDM. (TIF)

S1 Table PHMB:CpG ODN and PHMB concentrations presented in various units. (a) Shows the concentration of free PHMB and (b-d) show concentrations of the polyplex for each PHMB to CpG ODN ratio.
(PDF)

S2 Table Measurements of IL-4 and TNF-α production. Calculated concentrations of (a) IL-4 and (b) TNF-α production in ng/ml after treated with PHMB (rows 3 and 4), CpG ODN (rows 5 and 6) or PHMB/CpG ODN polyplexes (rows 7 and 8) at different concentrations. Rows 1 and 2 are serially diluted (1:1) standards. Rows 9A and 10A are controls.
(TIF)
