# Supplementary figures and images for: Pathogen- and Host-Directed Antileishmanial Effects Mediated by Polyhexanide (PHMB)
Source: PLoS Negl Trop Dis. 2015 Oct 2;9(10):e0004041. doi: 10.1371/journal.pntd.0004041 (PMC4592236; doi:10.1371/journal.pntd.0004041)

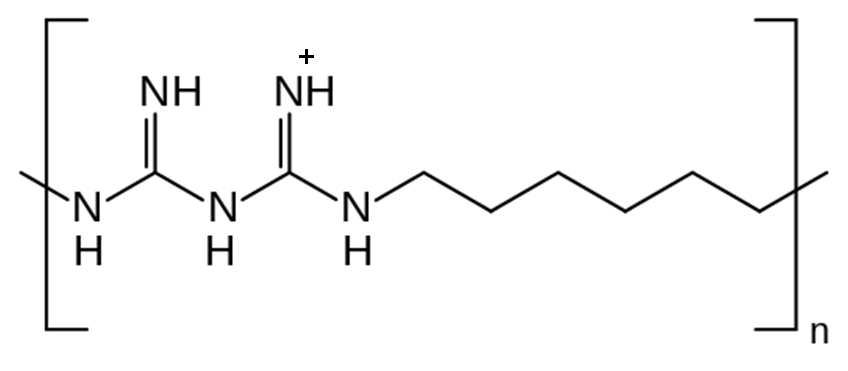

Supplement: S1 Fig — (TIF) [file pntd.0004041.s001.tif]

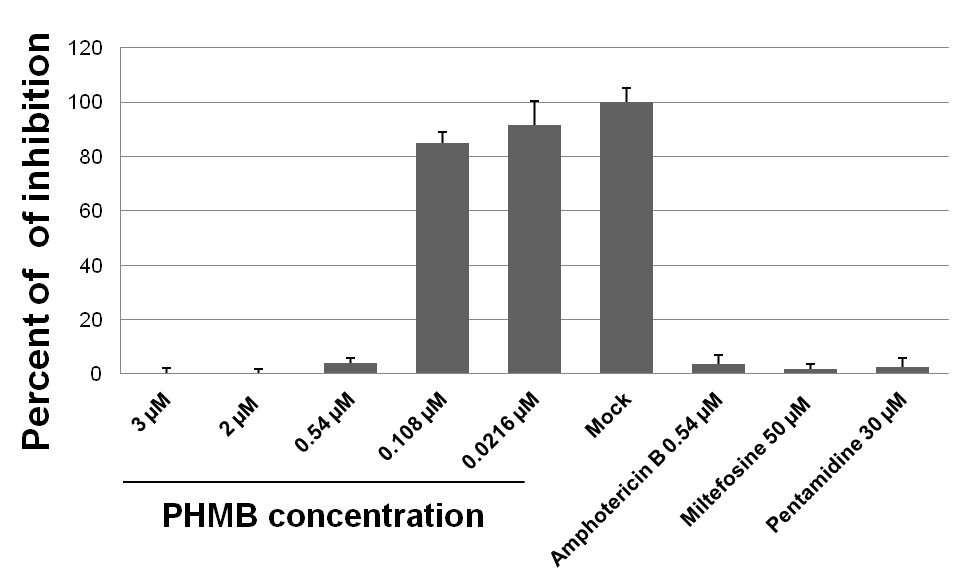

Supplement: S2 Fig — The error bars show the standard error of three independent experiments. (TIF) [file pntd.0004041.s002.tif]

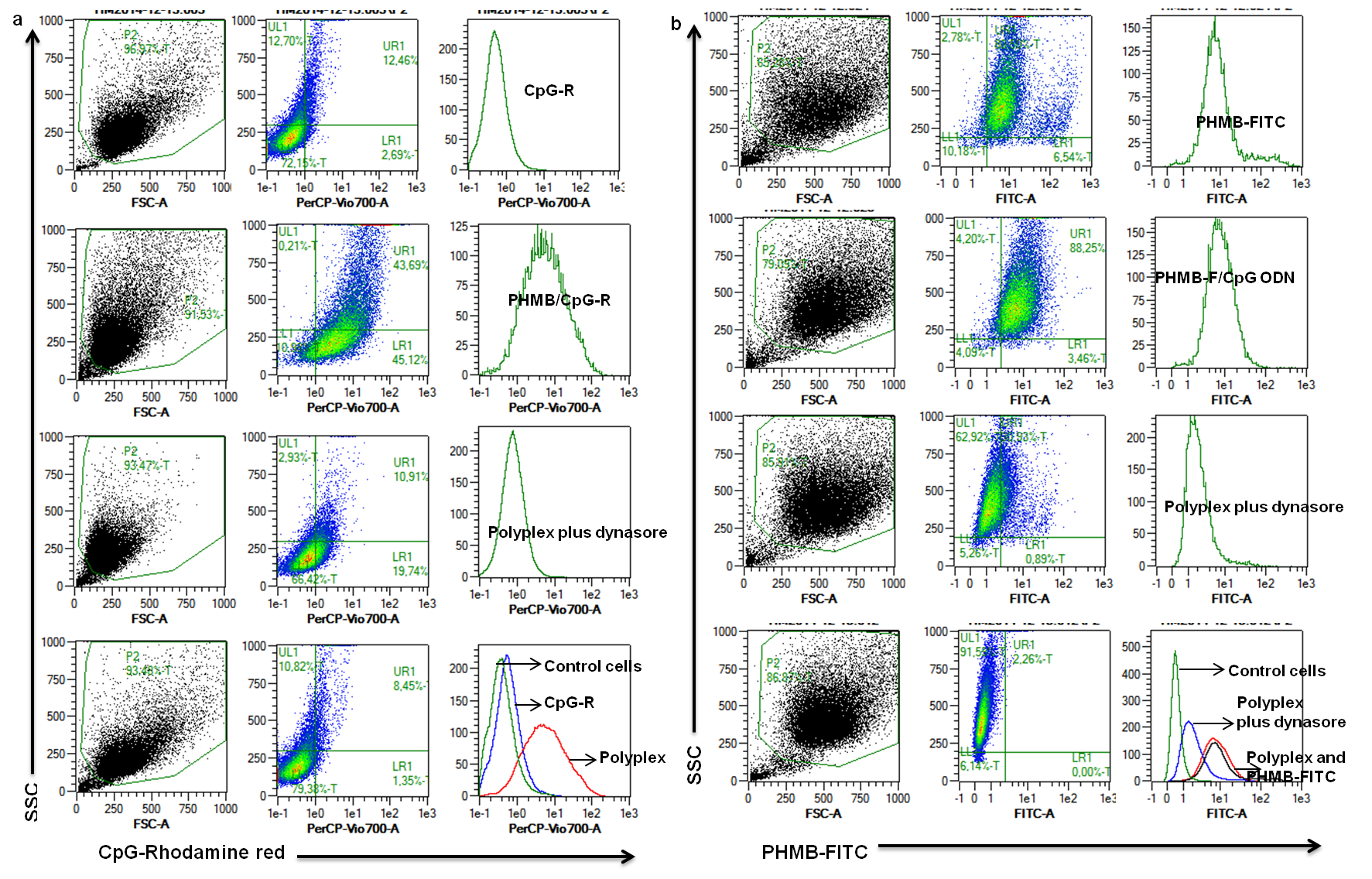

Supplement: S3 Fig — The cellular uptake potential of (a) PHMB/CpG-R and (b) PHMB-FITC/CpG ODN polyplexes, and inhibition by dynasore. Free PHMB-FITC, PHMB-FITC/CpG ODN and PHMB/CpG-R polyplexes were efficiently blocked by dynasore. Based on their MFI, the uptake of CpG-R was enhanced by about 15 folds as polyplex form compared to its free form. (TIF) [file pntd.0004041.s003.tif]

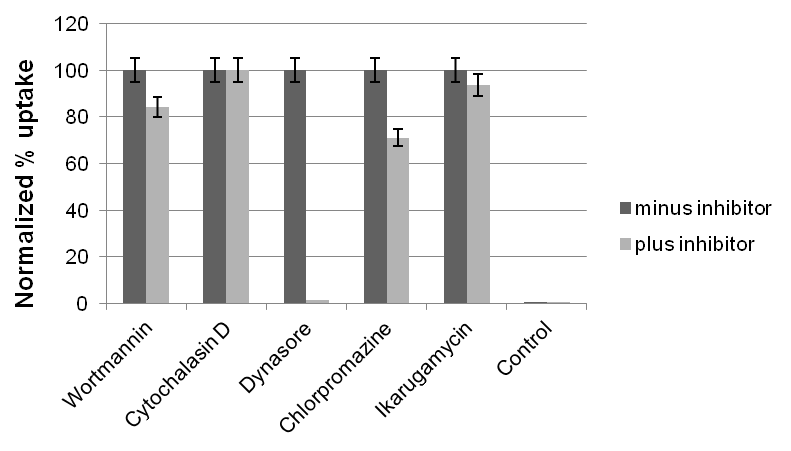

Supplement: S4 Fig — The bar graphs show the effects of different inhibitors on blocking the uptake of PHMB-FITC by promastigotes. Normalized mean fluorescence intensity (MFI) values of three independent flow cytometry experiments are depicted as mean ± SE. (TIF) [file pntd.0004041.s004.tif]

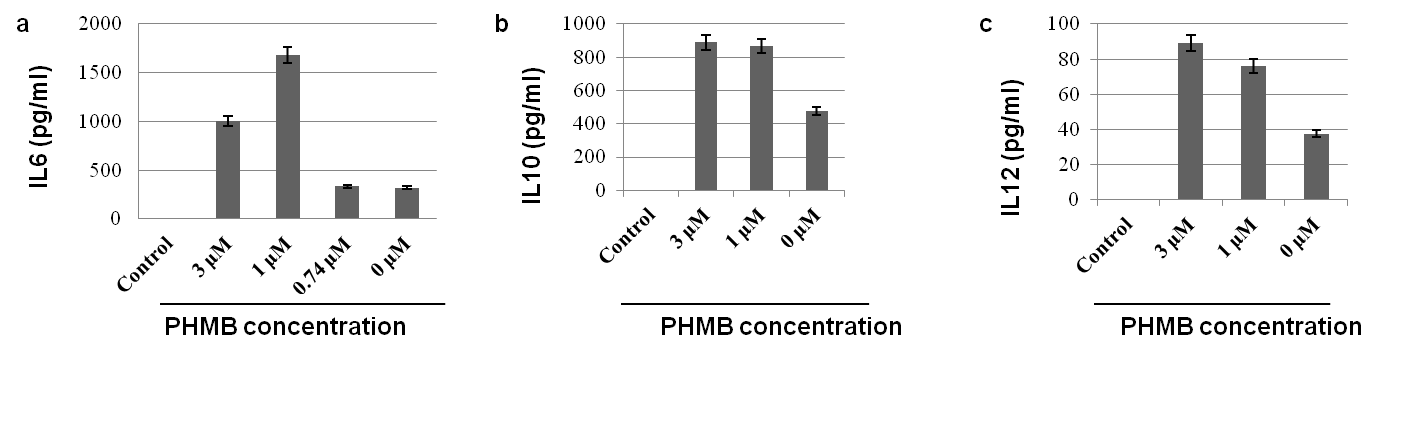

Supplement: S5 Fig — (TIF) [file pntd.0004041.s005.tif]

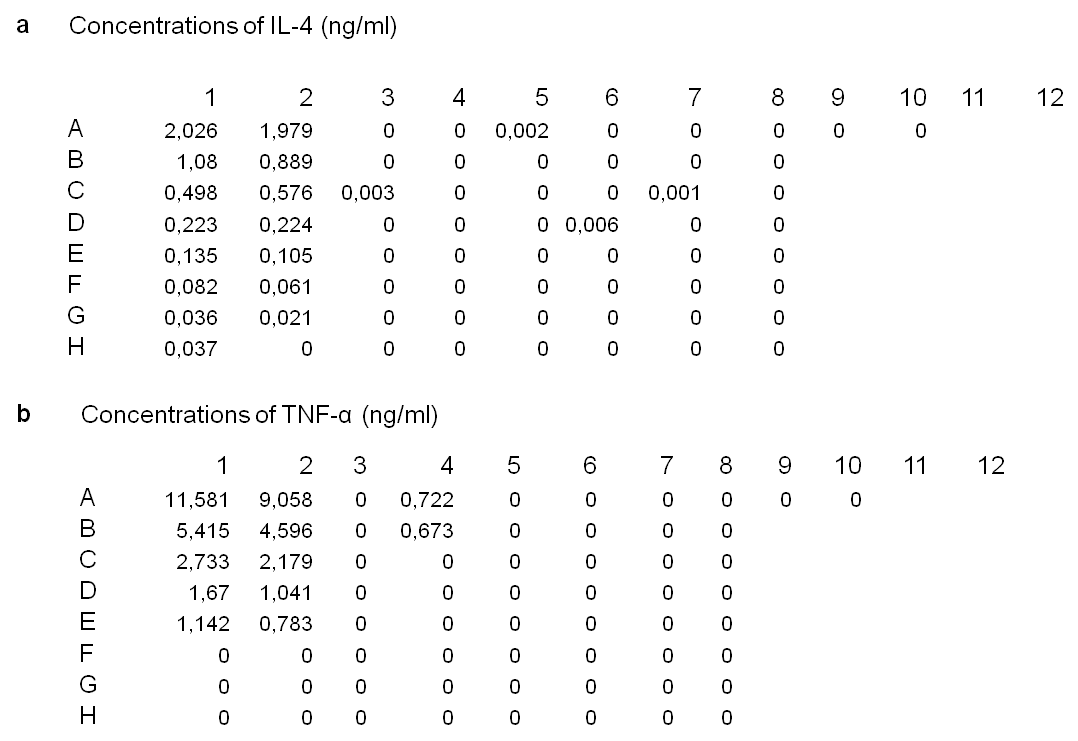

Supplement: S2 Table — Calculated concentrations of (a) IL-4 and (b) TNF-α production in ng/ml after treated with PHMB (rows 3 and 4), CpG ODN (rows 5 and 6) or PHMB/CpG ODN polyplexes (rows 7 and 8) at different concentrations. Rows 1 and 2 are serially diluted (1:1) standards. Rows 9A and 10A are controls. (TIF) [file pntd.0004041.s007.tif]
