# Supplementary material for: Pathogen- and Host-Directed Antileishmanial Effects Mediated by Polyhexanide (PHMB)
Source: PLoS Negl Trop Dis. 2015 Oct 2;9(10):e0004041. doi: 10.1371/journal.pntd.0004041 (PMC4592236; doi:10.1371/journal.pntd.0004041)
Supplement: S1 Table — (a) Shows the concentration of free PHMB and (b-d) show concentrations of the polyplex for each PHMB to CpG ODN ratio. (PDF) [file pntd.0004041.s006.pdf]

Supplementary Table 1. PHMB:CpG ODN and PHMB concentrations presented in various units.

a. PHMB alone

IC 50	**IC50 (μM)**	**IC50 (μg/ml)**	**% (w/v)**
Promastigotes	0.41 ± 0.25	1.23±0.75	0.0001%
Amastigotes	4 ± 0.65	12±1.95	0.001%
Epithelial cells	26	78	0.008%
Keratinocytes	6.6±3.5	19.8±10.5	0.002%
BMDM	4± 0.7	12±2.1	0.001%

b. PHMB/CPG (1:2 w/w; 1:0.34 N/P)

	**IC_{50} (μM)**	**IC_{50} (μg/ml)**	**% (w/v)**
Promastigotes	2.54 ± 0.5	7.62±1.5	0.0008%
BMDM	134 ± 40.2	402±120.6	0.04%
Amastigotes	1.12 ± 0.23	3.36±0.69	0.0003%

c. PHMB/CPG (1:1 w/w; 1:0.17 N/P)

	**IC_{50} (μM)**	**IC_{50} (μg/ml)**	**% (w/v)**
Promastigotes	2.2 ± 0.28	6.6±0.84	0.0007%
BMDM	50 ± 10.4	150±31.2	0.02%
Amastigotes	2. 82 ± 2.8	8.4±8.4	0.0008%

d. PHMB/CpG (2:1 w/w; 1:0.09 N/P)

	**IC_{50} (μM)**	**IC_{50} (μg/ml)**	**% (w/v)**
Promastigotes	1.45± 0.33	4.35± 0.33	0.0004%
BMDM	33 ± 9.5	99± 28.5	0.01%
Amastigotes	1± 0.55	3± 1.65	0.0004%
